# Supplementary material for: Genetically personalised organ-specific metabolic models in health and disease
Source: Nat Commun. 2022 Nov 29;13:7356. doi: 10.1038/s41467-022-35017-7 (PMC9708841; doi:10.1038/s41467-022-35017-7)
Supplement: Supplementary file 1 — Supplementary Information [file 41467_2022_35017_MOESM1_ESM.pdf]

Supplementary Figures

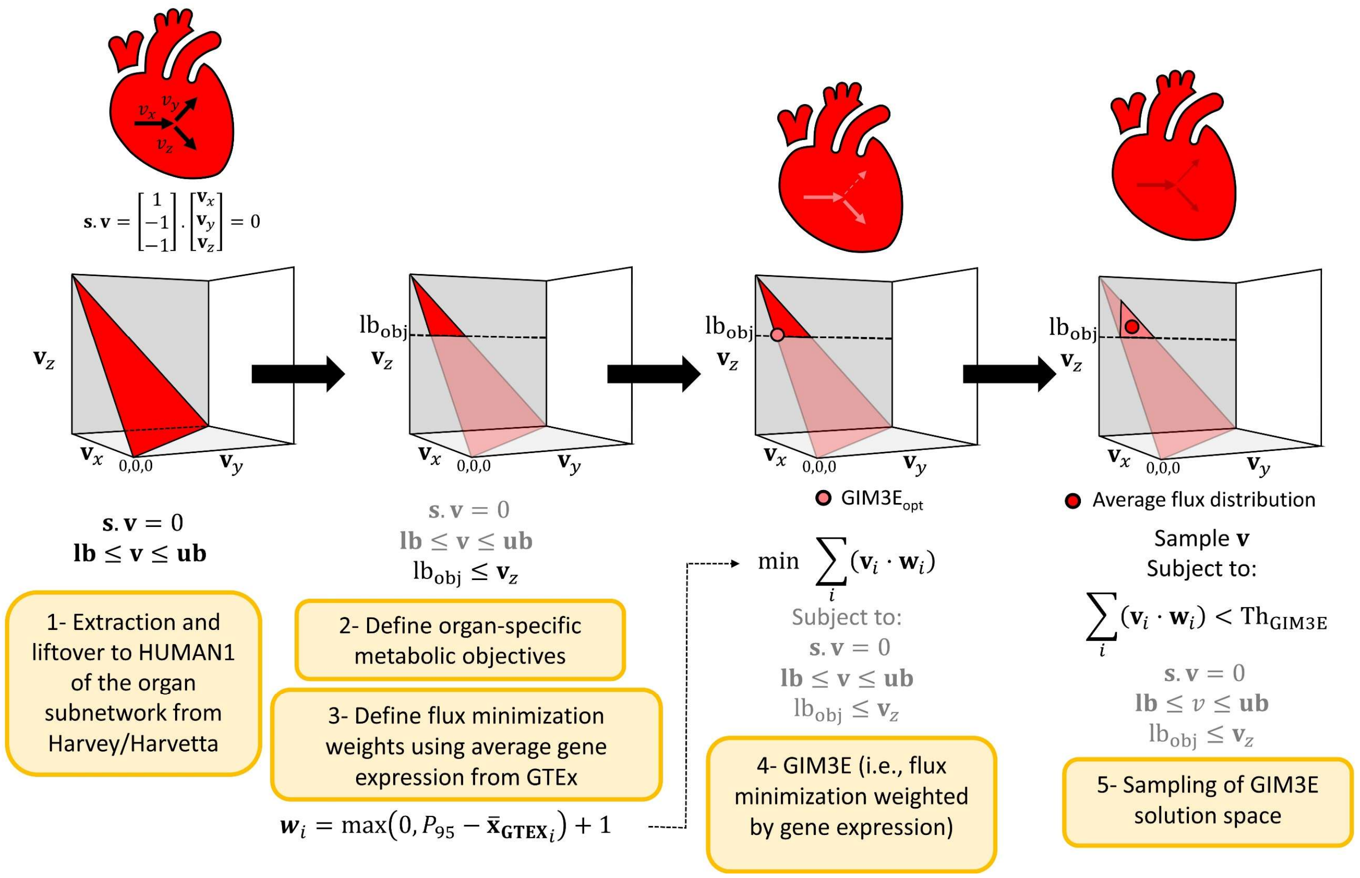

Supplementary Figure 1: Computing the organ-specific reference flux distribution. First, we extract the organ-subnetwork from the Harvey/Harvetta multiorgan models and we perform a liftover to update it to HUMAN1. From this subnetwork, an organ-specific solution space feasible with the reaction stoichiometry in each organ is defined. **s** is the stoichiometric matrix, **v** is a vector of steady-state flux values and **lb** and **ub** are the vector of reaction lower and upper bounds, respectively. An example of the stoichiometric matrix for a reaction network with three reactions *(x,y* and *z)* and one ramification is provided, and a hypothetical feasible space is shown in a 3D space. Next, a set of organ-specific metabolic objectives are defined, and the solution space is constrained to only solutions above a given threshold (lb_{obj}) for these objectives. Next, a vector of minimisation weights (**w**) for reactions is defined using average transcript abundances from GTEx mapped to reactions of the organ-specific network ($\bar{\mathbf{x}}_{\mathbf{GTEx}}$). P_{95} is the 95th percentile of the average transcript abundance values mapped to reactions of the organ-specific network. Then, the GIM3E algorithm is used to perform a weighted minimisation of total reaction fluxes. Finally, the vicinity of the GIM3E solution space is defined and sampled to obtain a representative flux map. Th_{GIM3E} is the maximum value of the GIM3E objective used to define the vicinity of the GIM3E solution space.

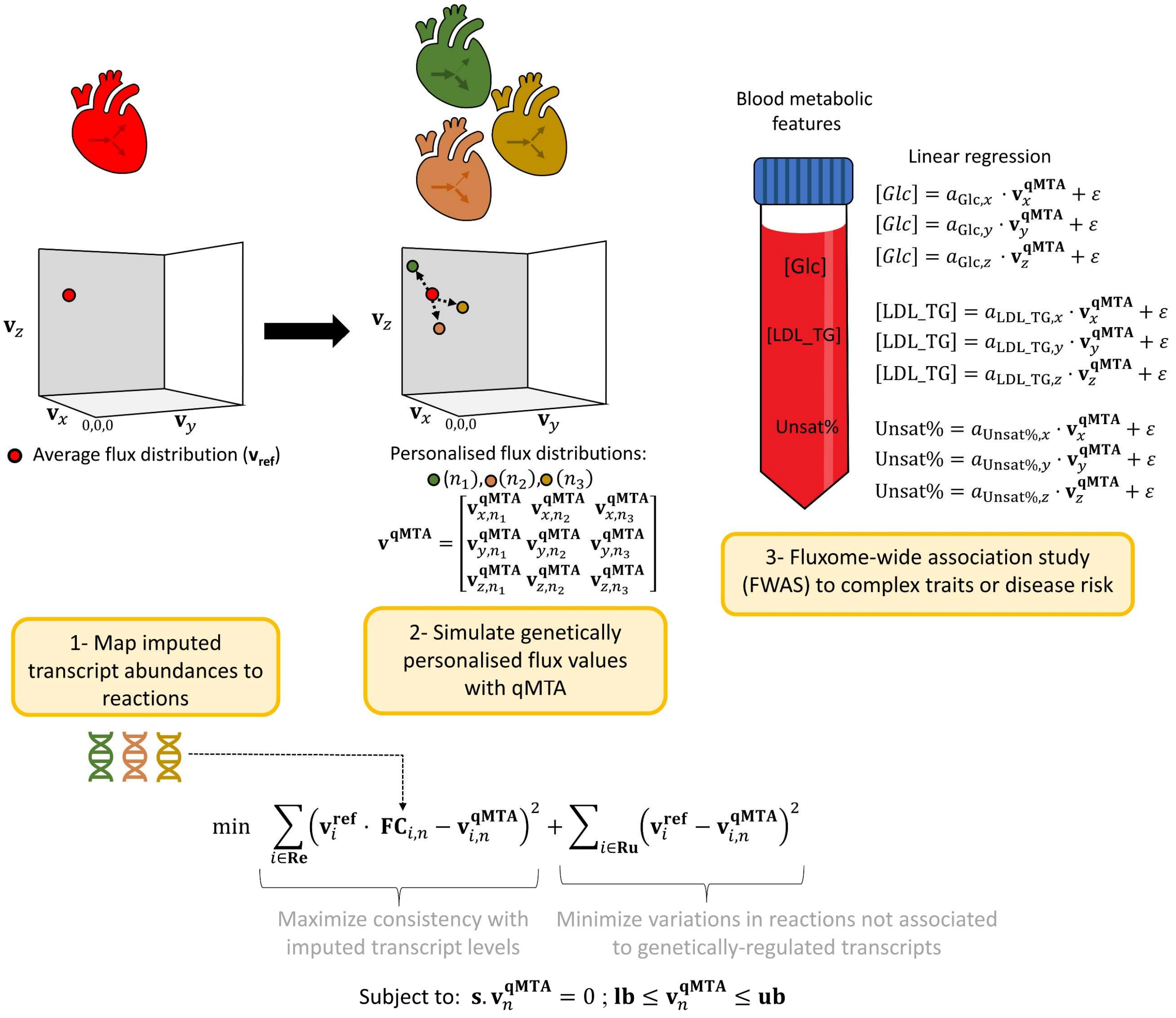

Supplementary Figure 2: Genetically personalised flux values and fluxome-wide association studies (FWAS). First, personalised organ-specific transcript abundances are imputed from genotype data and mapped to reactions in the organ-specific metabolic network as reaction activity fold changes. Next, the quadratic metabolic transformation algorithm (qMTA) is used to find the flux distribution most consistent with the reaction activity fold changes in each individual, starting from the reference flux distribution. $\mathbf{v}^{\mathbf{ref}}$ is the flux vector of the reference flux distribution. **Re** are reactions mapped to imputed gene expression. **Ru** are reactions not mapped to imputed gene expression. $\mathbf{FC}_{i,n}$ is the reaction activity fold change imputed from genotype data for reaction *i* in individual *n*. $v_{i,n}^{qMTA}$ is the flux value for reaction *i* computed for individual *n*. v_n^{qMTA} is the flux vector computed for individual *n*. $\mathbf{s}$ is the stoichiometric matrix. **lb** and **ub** are the vector of reaction lower and upper bounds, respectively. For clarity, some parameters have been omitted from the qMTA equation; the complete equation can be found in Methods. The resulting personalised flux values can be used to perform FWAS on complex traits or diseases. As part of this process, a given trait is independently regressed against the fluxes through each reaction in the network to test for association. For instance, to perform FWAS on the blood metabolome, the measure of each metabolite will be linearly regressed against the flux through all the independent reactions in a given organ metabolic network. Blood metabolic features can include the concentration of metabolites (e.g., [Glc]: Glucose concentration), the concentration of metabolite fractions in lipoproteins (e.g. [LDL_TG]: Triglycerides in LDL) and ratios and relative measures (e.g., Unsat%: degree of fatty acid unsaturation). $a_{\mathrm{Met},i}$ is the effect size of flux *i* on metabolite Met.

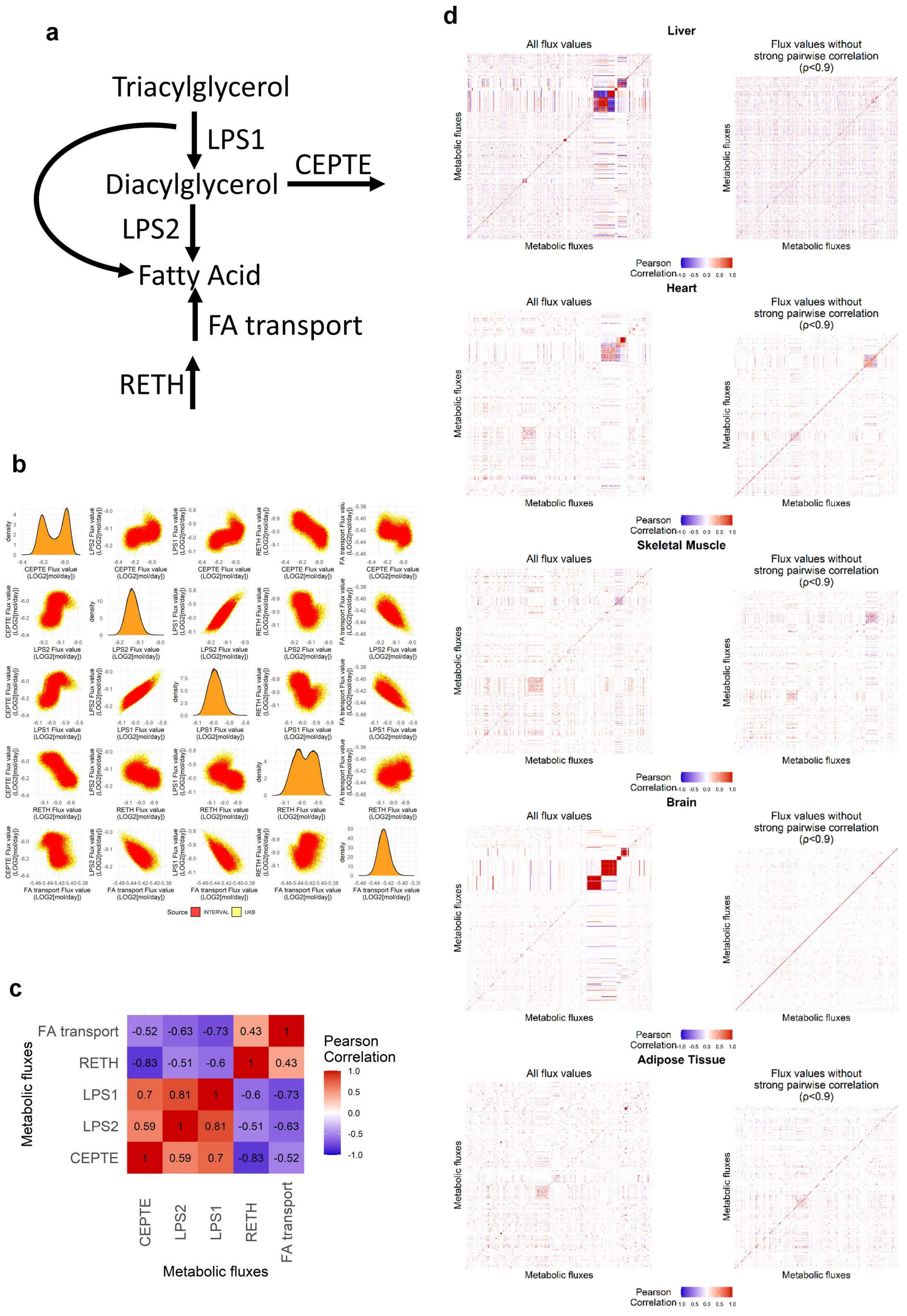
a
Triacylglycerol
LPS1
Diacylglycerol
CEPTE
LPS2
Fatty Acid
FA transport
RETH
b
CEPTE Flux value (LOG2[mol/day])
LPS2 Flux value (LOG2[mol/day])
LPS1 Flux value (LOG2[mol/day])
RETH Flux value (LOG2[mol/day])
FA transport Flux value (LOG2[mol/day])
density
Source INTERVAL UKB
c
FA transport -0.52 -0.63 -0.73 0.43 1
RETH -0.83 -0.51 -0.6 1 0.43
LPS1 0.7 0.81 1 -0.6 -0.73
LPS2 0.59 1 0.81 -0.51 -0.63
CEPTE 1 0.59 0.7 -0.83 -0.52
CEPTE LPS2 LPS1 RETH FA transport
Metabolic fluxes
Pearson Correlation
1.0
0.5
0.0
-0.5
-1.0
d
Liver
Heart
Skeletal Muscle
Brain
Adipose Tissue
All flux values
Flux values without strong pairwise correlation (ρ<0.9)
Metabolic fluxes
Pearson Correlation

Supplementary Figure 3: Correlation between genetically personalised fluxes. (a,b,c) Example of correlation between fluxes in a set of reactions in the liver. (a) Set of interconnected reactions centred around triacylglycerol in the liver. (b) Density plots and scatter plots for genetically personalised flux values through the reactions in the pathway. (c) Heatmap of pairwise Pearson correlation coefficients between reaction flux values in the set of reactions. Stoichiometric coupling between reactions (i.e., reactions sharing substrates or products) can result in correlations between the fluxes of the coupled reactions. CEPTE: Ethanolamine Phosphotransferase; FA transport: transport of fatty acids into hepatocytes; LPS: Triacylglycerol lipase; LPS2: Diacylglycerol lipase; RETH: Retinyl ester hydrolase. (d) Heatmaps of pairwise Pearson correlation coefficients between all reaction flux values in each organ before and after filtering out reactions with strong pairwise correlations. Reaction fluxes in each organ are ordered based on the hierarchical clustering of correlation coefficients. To remove reaction flux pairs with strong correlation, for each pair of reaction flux values with $\rho>0.9$, the feature with the largest mean absolute correlation to other flux values was removed.

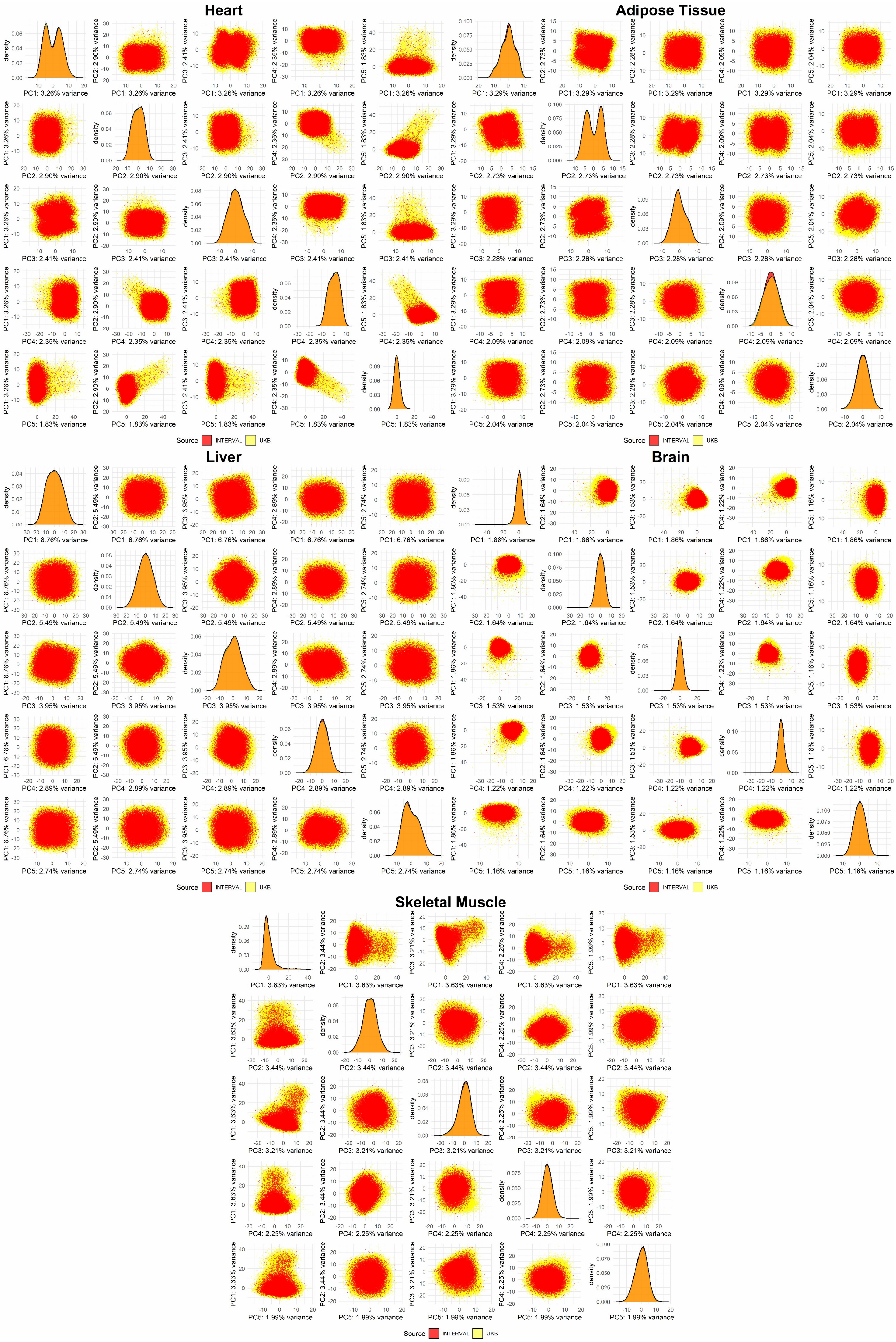
Heart
Adipose Tissue
Liver
Brain
Skeletal Muscle
PC1: 3.26% variance
PC2: 2.90% variance
PC3: 2.41% variance
PC4: 2.35% variance
PC5: 1.83% variance
PC1: 3.29% variance
PC2: 2.73% variance
PC3: 2.28% variance
PC4: 2.09% variance
PC5: 2.04% variance
PC1: 6.76% variance
PC2: 5.49% variance
PC3: 3.95% variance
PC4: 2.89% variance
PC5: 2.74% variance
PC1: 1.86% variance
PC2: 1.64% variance
PC3: 1.53% variance
PC4: 1.22% variance
PC5: 1.16% variance
PC1: 3.63% variance
PC2: 3.44% variance
PC3: 3.21% variance
PC4: 2.25% variance
PC5: 1.99% variance
density
Source
INTERVAL
UKB

Supplementary Figure 4: Principal component analysis. Pairwise score plots between the first five principal components in UKB and INTERVAL computed using genetically personalised metabolic flux values as features. Each organ is analysed and plotted independently.

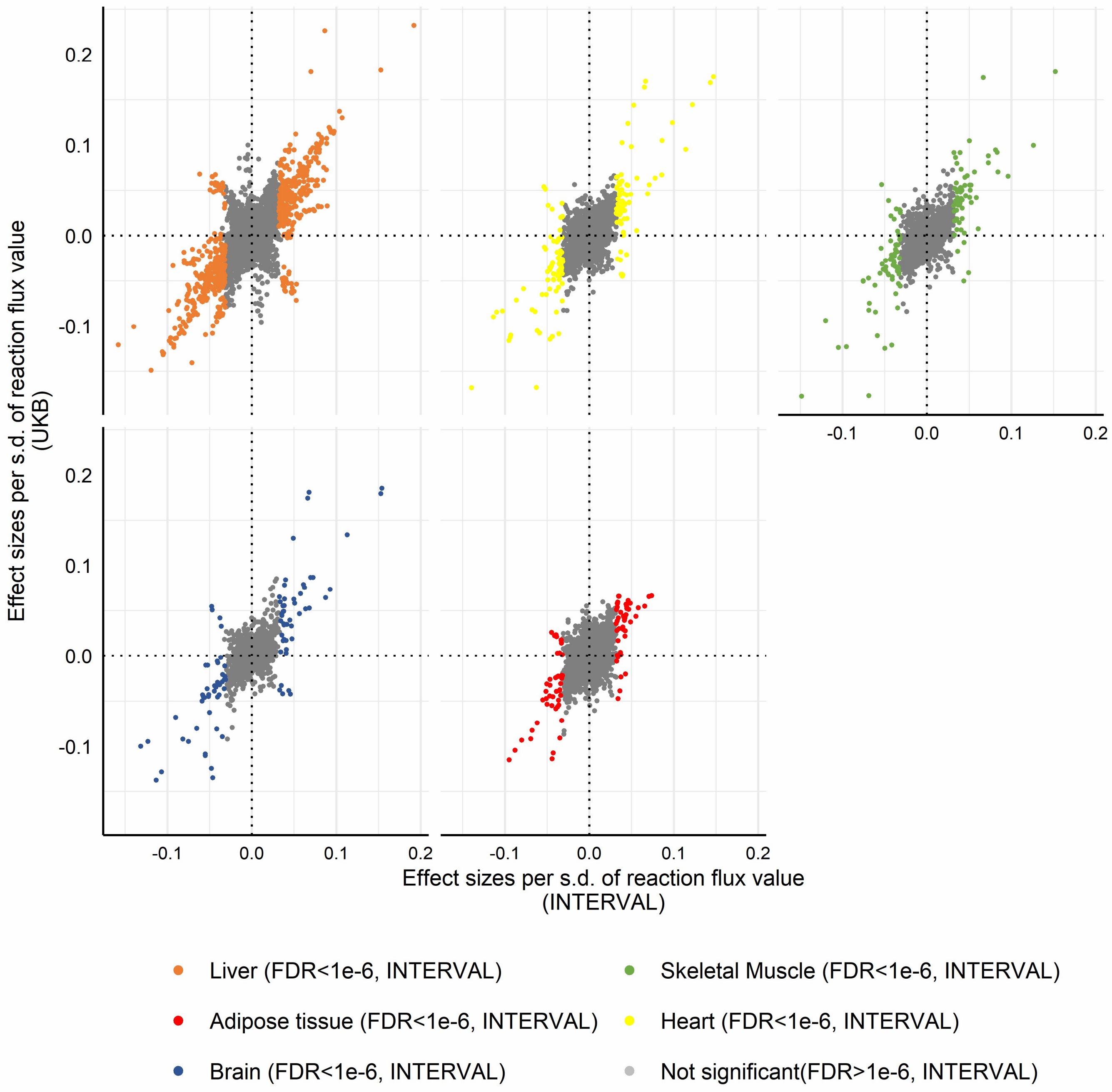

Supplementary Figure 5: Plot of flux effect sizes per organ to blood metabolic features measured with the Nightingale Health platform in INTERVAL and UK Biobank. Statistically significant effect sizes (FDR-adjusted P-value<10-6) in the INTERVAL cohort are highlighted. The statistical significance of each flux to blood metabolic feature associations was evaluated with linear regression (two-tailed t-test for flux effect size; Methods).

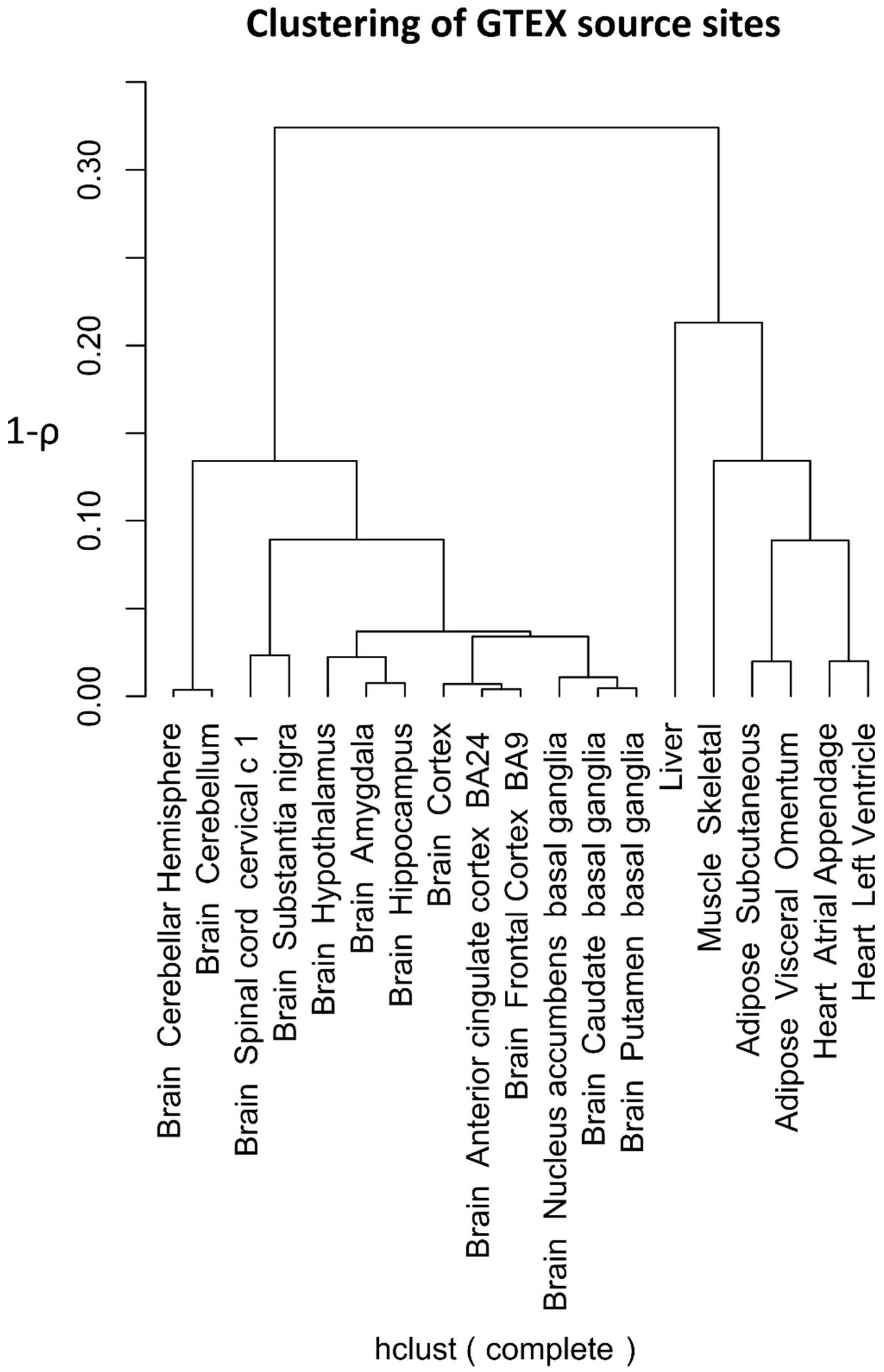

Supplementary Figure 6: Clustering of GTEx source sites. Source sites are clustered hierarchically using the correlation between average transcripts abundance in each site as a measure of similarity.
