# Supplementary material for: Genetically personalised organ-specific metabolic models in health and disease
Source: Nat Commun. 2022 Nov 29;13:7356. doi: 10.1038/s41467-022-35017-7 (PMC9708841; doi:10.1038/s41467-022-35017-7)
Supplement: Supplementary file 3 — Description of Additional Supplementary Files [file 41467_2022_35017_MOESM3_ESM.pdf]

Description of Additional Supplementary Files

File Name: Supplementary Data 1
Description: Principal component loadings of genetically personalised flux values across all modelled organs. The ten flux values with the most contribution to the first five principal components are indicated. Each organ is analysed independently.

File Name: Supplementary Data 2
Description: FWAS to blood metabolic features. A table detailing the significant associations (FDR-adjusted P-value$<10^{-6}$) identified in the INTERVAL cohort between genetically personalised flux values and blood metabolic features. The statistical significance of each flux to blood metabolic feature associations was evaluated with linear regression (two-tailed t-test for flux effect size; Methods). Significant associations identified with genetically personalised flux values computed with Recon3D-based models are also provided.

File Name: Supplementary Data 3
Description: Extended FWAS to lipoprotein fractions in the liver. Once FWAS analysis had established the importance of liver fluxes in lipoprotein fractions, FWAS was repeated using all fluxes from the liver (i.e., without excluding fluxes with strong intercorrelation), and all lipoproteins blood features (i.e., without excluding features with strong intercorrelation) to provide a complete picture. The table details the 8,152 significant (FDR-adjusted P-value$<10^{-6}$) associations in the INTERVAL cohort between all genetically personalised flux values in the liver and all lipoprotein features. The statistical significance of each flux to lipoprotein associations was evaluated with linear regression (two-tailed t-test for flux effect size; Methods). The significance of the same associations computed with Recon3D-based models is also indicated.

File Name: Supplementary Data 4
Description: FWAS to coronary artery disease. Table detailing the 92 genetically personalised flux values with significant (FDR-adjusted P-value<0.05) associations to coronary artery disease risk in the UK Biobank cohort. Statistical significance of the association to each flux to coronary artery disease was evaluated with a Cox proportional hazards regression (two-tailed Wald test for flux hazard ratios; Methods). The significance of the same associations computed with Recon3D-based models is also indicated.

File Name: Supplementary Data 5
Description: Tasks used for model validation. Table detailing essential and organ-specific metabolic tasks that must be feasible in organ-specific metabolic networks. They are defined using the HUMAN1 format for metabolic tasks.

File Name: Supplementary Data 6
Description: Metabolic objectives for each organ. Organ-specific metabolic objectives represent major metabolic functions that the organ must fulfil in the condition of study. They are used to compute the reference flux distribution using the GIM3E algorithm.
