# Supplementary material for: Genetically personalised organ-specific metabolic models in health and disease
Source: Nat Commun. 2022 Nov 29;13:7356. doi: 10.1038/s41467-022-35017-7 (PMC9708841; doi:10.1038/s41467-022-35017-7)
Supplement: Supplementary file 10 — Reporting Summary [file 41467_2022_35017_MOESM10_ESM.pdf]

nature portfolio

Corresponding author(s): Carles Foguet, Michael Inouye

Last updated by author(s): Nov 3, 2022

Reporting Summary

Nature Portfolio wishes to improve the reproducibility of the work that we publish. This form provides structure for consistency and transparency in reporting. For further information on Nature Portfolio policies, see our Editorial Policies and the Editorial Policy Checklist.

Statistics

For all statistical analyses, confirm that the following items are present in the figure legend, table legend, main text, or Methods section.

n/a	Confirmed	
☐	☒	The exact sample size (*n*) for each experimental group/condition, given as a discrete number and unit of measurement
☐	☒	A statement on whether measurements were taken from distinct samples or whether the same sample was measured repeatedly
☐	☒	The statistical test(s) used AND whether they are one- or two-sided<br>*Only common tests should be described solely by name; describe more complex techniques in the Methods section.*
☐	☒	A description of all covariates tested
☐	☒	A description of any assumptions or corrections, such as tests of normality and adjustment for multiple comparisons
☐	☒	A full description of the statistical parameters including central tendency (e.g. means) or other basic estimates (e.g. regression coefficient) AND variation (e.g. standard deviation) or associated estimates of uncertainty (e.g. confidence intervals)
☐	☒	For null hypothesis testing, the test statistic (e.g. *F*, *t*, *r*) with confidence intervals, effect sizes, degrees of freedom and *P* value noted<br>*Give P values as exact values whenever suitable.*
☒	☐	For Bayesian analysis, information on the choice of priors and Markov chain Monte Carlo settings
☒	☐	For hierarchical and complex designs, identification of the appropriate level for tests and full reporting of outcomes
☐	☒	Estimates of effect sizes (e.g. Cohen's *d*, Pearson's *r*), indicating how they were calculated

Our web collection on statistics for biologists contains articles on many of the points above.

Software and code

Policy information about availability of computer code

Data collection

No specific software was used to collect the data.

Data analysis

The code used to generate personalised organ-specific flux maps from imputed gene expression data is available on GitHub (https://github.com/cfoguet/cobrafunctions) and permanently archived by Zenodo at https://doi.org/10.5281/zenodo.7277058. The code was run with python 2.7.5 using CPLEX 2.6 as a quadratic solver.
The following software and versions were also used:
-PLINK v2.00a3LM AVX2 Intel
- Python 3.6.8 along with python packages:
  - pandas 1.1.5
  - sklearn 0.24.2
  - lifelines 0.26.3
- R version 3.3.3 along with packages:
  - caret 6.0-86
  - dplyr 1.0.6
  - data.table 1.14.2
  - gaston 1.5.7
  - ggplot2 3.3.5
  - MASS 7.3-45
  - stringr 1.4.0
  - RSQLite_2.2.18

- ukbnmr 0.3.0

For manuscripts utilizing custom algorithms or software that are central to the research but not yet described in published literature, software must be made available to editors and reviewers. We strongly encourage code deposition in a community repository (e.g. GitHub). See the Nature Portfolio guidelines for submitting code & software for further information.

Data

Policy information about availability of data

All manuscripts must include a data availability statement. This statement should provide the following information, where applicable:

- Accession codes, unique identifiers, or web links for publicly available datasets
- A description of any restrictions on data availability
- For clinical datasets or third party data, please ensure that the statement adheres to our policy

The data from the INTERVAL and UK Biobank cohorts is under restricted access as it contains potentially identifying and sensitive patient information. It can be accessed by making a reasoned request to the INTERVAL coordination centre (https://www.intervalstudy.org.uk) and UKB (https://www.ukbiobank.ac.uk/), respectively. Response times from the data access committees are typically under one month. The summary statistics for the FWAS to blood metabolic features and CAD are provided in the Supplementary Data. The organ-specific genome-scale metabolic models generated in this work are available on the cobrafunctions GitHub repository (https://github.com/cfoguet/cobrafunctions), which is permanently archived by Zenodo (https://doi.org/10.5281/zenodo.7277058). HUMAN1 (version 1.11.0) can be obtained from the Human-GEM GitHub repository (https://doi.org/10.5281/zenodo.5992296). The Harvey and Harvetta models (1_03c) are available in the Supporting Information of reference 30. The elastic net PredictDB models (GTEx v8) models are available at https://predictdb.org. The GTEx gene expression data (GTEx Analysis Release V8; dbGaP Accession phs000424.v8.p2) can be obtained from https://gtexportal.org.

Human research participants

Policy information about studies involving human research participants and Sex and Gender in Research.

Reporting on sex and gender	In the manuscript, we use the term sex to describe the biological attribute. Sex was reported at the time of enrolment to UK Biobank or INTERVAL. Based on the reported sex, in the INTERVAL cohort, there are 22685 females and 22356 males. In the UKB cohort, there are 273353 females and 229106 males. We do not share individual-level data. The reported flux-phenotype associations apply to all sexes. Sex was used as a covariate in the regression of blood metabolites against flux values. In the COX regression model (association to coronary artery disease risk), samples were also stratified by sex.
Population characteristics	INTERVAL is a cohort of approximately 50,000 participants nested within a randomised trial studying the safety of varying the frequency of blood donation (ISRCTN24760606). Participants were blood donors aged 18 years and older (median 44 years of age; 50% women) . UK Biobank is a cohort of approximately 500,000 participants from the general UK population. Participants were between age 40 and 69 at recruitment (median 58 years of age; 54% women). Age, sex, principal components of genetic ancestry, body mass index, smoking status were used as covariates. Blood metabolite levels and ICD codes related to coronary artery disease were also collected and used in this work.
Recruitment	Interval participants were blood donors aged 18 years and older recruited between 2012 and 2014 from 25 NHS Blood and Transplant centers. UK Biobank participants were between age 40 and 69 at recruitment (median 58 years of age; 54% women) and accepted an invitation to attend one of the assessment centres that were established across the United Kingdom between 2006 and 2010.
Ethics oversight	INTERVAL was approved by the National Research Ethics Service (11/EE/0538). UK Biobank has approval from the North West Multi-centre Research Ethics Committee (MREC) as a Research Tissue Bank (RTB). Access to UK Biobank data was granted by UK Biobank under application ID 7439.

Note that full information on the approval of the study protocol must also be provided in the manuscript.

Field-specific reporting

Please select the one below that is the best fit for your research. If you are not sure, read the appropriate sections before making your selection.

☒ Life sciences ☐ Behavioural & social sciences ☐ Ecological, evolutionary & environmental sciences

For a reference copy of the document with all sections, see nature.com/documents/nr-reporting-summary-flat.pdf

Life sciences study design

All studies must disclose on these points even when the disclosure is negative.

Sample size	Sample size was determined by the available data in the INTERVAL (N=37220) and UK Biobank (N=487,395) cohorts, excluding measures that did not pass quality control. No sample size calculations were performed in this work, but such sample sizes have proven to be more than adequate in numerous previous studies using such cohorts.
Data exclusions	Only measures that did not pass quality control were excluded.

Replication	FWAS (Fluxome Wide Association Study) inherently includes intra-cohort replication as it identifies flux to phenotype associations shared by individuals (replicates) in a given cohort. FWAS to blood metabolic features was performed in 37220 individuals of the INTERVAL cohort and 120266 individuals of the UK Biobank. FWAS to coronary artery disease (CAD) risk was performed in 462790 individuals of UKB (34121 events of CAD and 428669 controls). Concerning inter-cohort replication, we found that 83% of the INTERVAL associations to blood metabolic features measured with the Nightingale Health platform could be replicated in UKB. We attribute the associations that could not be replicated to different cohort compositions as INTERVAL participants are, on average, both younger and healthier than UK Biobank participants. Associations to blood metabolic features measured by the Metabolon HD4 platform could not be replicated across cohorts as they were only measured in INTERVAL. Likewise, the association with CAD risk in UK Biobank could not be evaluated in INTERVAL due to the low number of CAD cases in this cohort.<br><br>All attempted replication experiments are reported here and in detail in the Main Text and Supplementary Material.
Randomization	Not applicable as we do not make use of experimental groups in our analyses.
Blinding	Not applicable as we use existing cohorts and we do not make use of predefined groups in our analyses.

Reporting for specific materials, systems and methods

We require information from authors about some types of materials, experimental systems and methods used in many studies. Here, indicate whether each material, system or method listed is relevant to your study. If you are not sure if a list item applies to your research, read the appropriate section before selecting a response.

Materials & experimental systems

n/a	Involved in the study
☒	☐ Antibodies
☒	☐ Eukaryotic cell lines
☒	☐ Palaeontology and archaeology
☒	☐ Animals and other organisms
☐	☒ Clinical data
☒	☐ Dual use research of concern

Methods

n/a	Involved in the study
☒	☐ ChIP-seq
☒	☐ Flow cytometry
☒	☐ MRI-based neuroimaging

Clinical data

Policy information about clinical studies

All manuscripts should comply with the ICMJE guidelines for publication of clinical research and a completed CONSORT checklist must be included with all submissions.

Clinical trial registration	INTERVAL (NCT01610635). Access to UK Biobank data was granted by UK Biobank under application ID 7439
Study protocol	INTERVAL: https://doi.org/10.1186/1745-6215-15-363 ; UK Biobank: https://doi.org/10.1371/journal.pmed.1001779
Data collection	In INTERVAL, participants were blood donors aged 18 years and recruited between 2012 and 2014 from 25 NHS Blood and Transplant centers. In UK Biobank, participants accepted an invitation to attend one of the assessment centers that were established across the United Kingdom between 2006 and 2010. In both INTERVAL and UKB, blood samples were predominantly collected and analyzed from the baseline visit. Anthropometric measurements and health and lifestyle questionnaires were also collected.
Outcomes	The outcome of this work is to identify associations between genetically personalized organ-specific flux values and phenotypes such as metabolite levels in the blood (measured with either the Nightingale or Metabolon platforms) or coronary artery disease (measured by reported ICD codes). We note that in this work, we make use of two existing cohorts (INTERVAL and UK Biobank) that were not originally designed for this purpose.
